# New and little known species of oribatid mites of the family Haplozetidae (Acari, Oribatida) from Ecuador

**DOI:** 10.3897/zookeys.346.6436

**Published:** 2013-11-01

**Authors:** Sergey G. Ermilov, Badamdorj Bayartogtokh, Dorothee Sandmann, Franca Marian, Mark Maraun

**Affiliations:** 1 Tyumen State University, Tyumen, Russia; 2 National University of Mongolia, Department of Zoology, Ulaanbaatar, Mongolia; 3 Georg-August-University Göttingen, J.F. Blumenbach Institute of Zoology and Anthropology, Göttingen, Germany

**Keywords:** Oribatida, Haplozetidae, new species, *Haplozetes*, *Protoribates*, supplementary description, *Trachyoribates* (*Rostrozetes*) *glaber* (Beck, 1965), Ecuador

## Abstract

We described two new species, *Haplozetes paraminimicoma* **sp. n.** and *Protoribates ecuadoriensis* **sp. n.** from Ecuador. Additionally, a detailed supplementary description of *Trachyoribates* (*Rostrozetes*) *glaber* (Beck, 1965) is given on the basis of Ecuadorian specimens, which was known previously only from Peru. An annotated checklist of all identified taxa of Haplozetidae from Ecuador is presented.

## Introduction

The present study is based on the oribatid mite materials collected from the tropical rain forest soils in Ecuador, between 2008 and 2010. This paper is part of our continuing studies (see Ermilov et al. 2013a–c), and it includes the data on the family Haplozetidae. An annotated checklist of identified taxa is presented below.

In the course of taxonomic identification of the haplozetid mites, we found two new species belonging to the genera *Haplozetes* Willman, 1935 and *Protoribates* Berlese, 1908.

The genus *Haplozetes* was proposed by Willmann (1935) with *Peloribates vindobonensis* Willmann, 1935 as the type species. Subsequently, Grandjean (1936) redefined the type species, and ascertained generic status of *Haplozetes* and proposed the family Haplozetidae. This status has been accepted later by many authors (e.g. Balogh 1963, 1965, 1972; Shaldybina 1975; Balogh and Balogh 1984, 1992; Pérez-Iñigo 1993; Bayartogtokh 2000; Weigmann 2006; Murvanidze and Weigmann 2012). Recently, Subías (2004) treated *Haplozetes* as a subgenus of *Indoribates* Jacot, 1929, without justifying this action. We do not agree with the latter author’s concept, and, therefore, consider here *Haplozetes* as an independent genus. This is one of the smallest genera of oribatid mites and currently comprises only 15 nominal species and one subspecies (Subías 2004, updated 2013).

The other genus studied here, *Protoribates* Berlese, 1908 is well defined by Weigmann et al. (1993), and is comparatively species rich as about 50 species were assigned to this genus (Subías 2004, updated 2013).

The third genus studied by us, *Trachyoribates* encompasses two subgenera, *Trachyoribates* Berlese, 1908 and *Rostrozetes* Sellnick, 1925, species of both of which are mainly distributed in the tropical regions (see Subías 2004, updated 2013).

The main purpose of our paper is to describe and illustrate two new species of *Haplozetes* and *Protoribates*. Also, a detailed supplementary description of *Trachyoribates (Rostrozetes) glaber* (Beck, 1965) is presented on the basis of Ecuadorian specimens.

## Materials and methods

The study materials are derived from the following two collecting sites:

Ec-1: Southern Ecuador, 3°70'S, 78°58'W, Bombuscaro, Podocarpus National Park, 1050 m a.s.l., upper organic soil layer in mostly undisturbed rain forest, 01.10.2008, 01.04.2009 and 01.08.2010, collected by F. Marian and D. Sandmann.

Ec-2: Southern Ecuador, 3°58'S, 79°50'W, Estación Científica San Francisco, 2000 m a.s.l., upper organic soil layer in mostly undisturbed rain forest, 01.09.2008 and 01.04.2009, collected by F. Marian and D. Sandmann.

Specimens were studied in lactic acid, mounted in temporary cavity slides for the duration of the study, and then stored in 70% ethanol in vials. Body length was measured in lateral view, from the tip of rostrum to the posterior edge of ventral plate. Notogastral width refers to the maximum width in dorsal aspect. Lengths of body setae were measured in lateral aspect. All body measurements are given in micrometers. General terminology used in this paper follows that summarized by Norton and Behan-Pelletier (2009).

### Checklist of identified Ecuadorian Haplozetidae

– *Haplozetes paraminimicoma* sp. n. Locality: Ec-1, Ec-2.
– *Protoribates iracemae* Pérez-Íñigo & Baggio, 1994. Locality: Ec-1. The species is recorded for the first time from Ecuador.
– *Protoribates paracapucinus* (Mahunka, 1988). Locality: Ec-1, Ec-2. The species is recorded for the first time from Ecuador and the Neotropical region.
– *Protoribates ecuadoriensis* sp. n. Locality: Ec-1, Ec-2.
– *Trachyoribates (Rostrozetes) glaber* (Beck, 1965). Locality: Ec-1. The species are recorded for the first time from Ecuador.

## Descriptions of new species

### Haplozetes paraminimicoma sp. n.

http://zoobank.org/F82D5CC5-9CCB-4B9B-B7EA-C3A03197634C

http://species-id.net/wiki/Haplozetes_paraminimicoma

Figs 1 , 2

#### Diagnosis.

Body size 332–348 × 215–249. Body surface smooth. Rostral and lamellar setae of medium long, with short cilia. Interlamellar setae short, smooth. Sensilli spindle-form, ciliate. Tutoria almost reaching of rostral margin, extending beyond level of insertions of rostral setae. Notogastral setae short, smooth. Genital plates with five pairs of setae. Epimeral, genital and aggenital setae with short cilia. Anal and adanal setae minute. Leg tarsi monodactylous. Leg tarsi I with 19 setae (*l*’’ absent).

#### Description.

*Measurements*. Body length: 348 (holotype), 332–348 (four paratypes); notogaster width: 232 (holotype), 215–249 (four paratypes).

*Integument*. Body color light brownish. Body surface smooth. Anterior part of pteromorphs striate.

*Prodorsum*. Rostrum rounded. Lamellae (*Lam*) located dorso-laterally, longer than half of prodorsum, reaching insertions of lamellar setae. Rostral (*ro*, 28–32) and lamellar (*le*, 32–36) setae setiform, with several short cilia. Interlamellar setae short (*in*, 8–12), thin, smooth. A pair of elongate, narrow porose areas *Ad* present latero-posterior to interlamellar setae (well visible in dissected specimen). Exobothridial setae (*ex*, 12–16) thin, with one or two cilia. Sensilli longest setae on prodorsum (*ss*, 94–106), spindle-form, with long stalk, lanceolate head and thin, point tip; distal part of stalk and sensillar head ciliate. Tutoria (*tu*) thin, almost straight, extending insertions of rostral setae, with small, free tooth (*t*) distally. Sublamellar lines (*Slam*) present, short, thin, poorly visible. Sublamellar porose areas (*Al*) small, rounded (4). Porose areas *Am* and *Ah* not observed.

*Notogaster*. Anterior notogastral margin convex medially. Dorsophragmata (*D*) and pleurophragmata (*P*) distinct. Pteromorphs sub-triangular. Ten pairs of notogastral setae short (6), thin, smooth. Four pairs of sacculi (*Sa*, *S1*, *S2*, *S3*) with small openings; *Sa* consisting of two adjacent parts, *S1* and *S2* irregular elongate oval, *S3* sub-triangular. Lyrifissures (*ia*, *im*, *ip*, *ih*, *ips*) and opisthonotal gland openings (*gla*) located typically for the genus (see Beck 1964; Bayartogtokh 2000). Postanal porose area not observed.

*Gnathosoma*. Subcapitulum longer than wide (82 × 61). Subcapitular setae setiform, with short cilia; *h* (12) shorter than *m* (28) and *a* (16). Two pairs of adoral setae (*or*_1_, *or*_2_, 12) setiform, straight, densely ciliate. Palps (69) with setation 0–2–1–3–9(+ω). Solenidion thickened, weakly dilated distally, coupled with eupathidium (*acm*). Chelicerae (82) with two setiform, ciliate setae; *cha* (28) longer and thicker than *chb* (18). Trägårdh’s organ (*Tg*) conical.

*Epimeral and lateral podosomal regions*. Apodemes 1, 2, 3 and sejugals well developed. Epimeral setal formula 3–2(1)–3–3; setae setiform, with short cilia. Setae *2b* present in holotype and two paratypes. Medial setae *1a*, *2a*, *3a* (8–10) shorter than others (16–20). Pedotecta I (*Pd* I), II (*Pd* II), discidia (*dis*) and circumpedal carinae (*cp*) developed typically for the genus (see Beck 1964; Bayartogtokh 2000).

*Anogenital region*. Five pairs of genital (*g*_1_–*g*_5_, 12) and one pair of aggenital (*ag*, 16) setae setiform, with short cilia. Two pairs of anal (*an*_1_, *an*_2_, 4) and three pairs of adanal (*ad*_1_–*ad*_3_, 4) setae minute. Lyrifissures *iad* in paraanal position.

*Legs*. All tarsi with one strong, dorsally weakly serrate claw. Morphology of leg segments, setae and solenidia typical for genus (see Beck 1964; Bayartogtokh 2000), hence only tarsus I is illustrated. Formulae of leg setation and solenidia: I (1–5–3–4–19) [1–2–2], II (1–5–3–4–15) [1–1–2], III (2–3–1–3–15) [1–1–0], IV (1–2–2–3–12) [0–1–0]; homology of setae and solenidia indicated in Table 1.

**Table 1.** Leg setation and solenidia of *Haplozetes paraminimicoma* sp. n.

Leg	Trochanter	Femur	Genu	Tibia	Tarsus
I	*v*’	*d*, *(l)*, *bv*’’, *v*’’	*(l)*, *v*’, σ	*(l)*, *(v)*, φ_1_, φ_2_	*(ft)*, *(tc)*, *(it)*, *(p)*, *(u)*, *(a)*, *s*, *(pv)*, *v*’, *(pl)*, *e*, ω_1_, ω_2_
II	*v*’	*d*, *l*_1_’, *l*_2_’, *bv*’’, *v*’’	*(l)*, *v*’, σ	*(l)*, *(v)*, φ	*(ft)*, *(tc)*, *(it)*, *(p)*, *(u)*, *(a)*, *s*, *(pv)*, ω_1_, ω_2_
III	*l*’, *v*’	*d*, *l*’, *ev*’	*l*’, σ	*l*’, *(v)*, φ	*(ft)*, *(tc)*, *(it)*, *(p)*, *(u)*, *(a)*, *s*, *(pv)*
IV	*v*’	*d*, *ev*’	*d*, *l*’	*l*’, *(v)*, φ	*ft*’’, *(tc)*, *(p)*, *(u)*, *(a)*, *s*, *(pv)*

Roman letters refer to normal setae (*e* to famulus), Greek letters to solenidia. Single prime (’) marks setae on anterior and double prime (’’) setae on posterior side of the given leg segment. Parentheses refer to a pair of setae.

**Figure 1.**
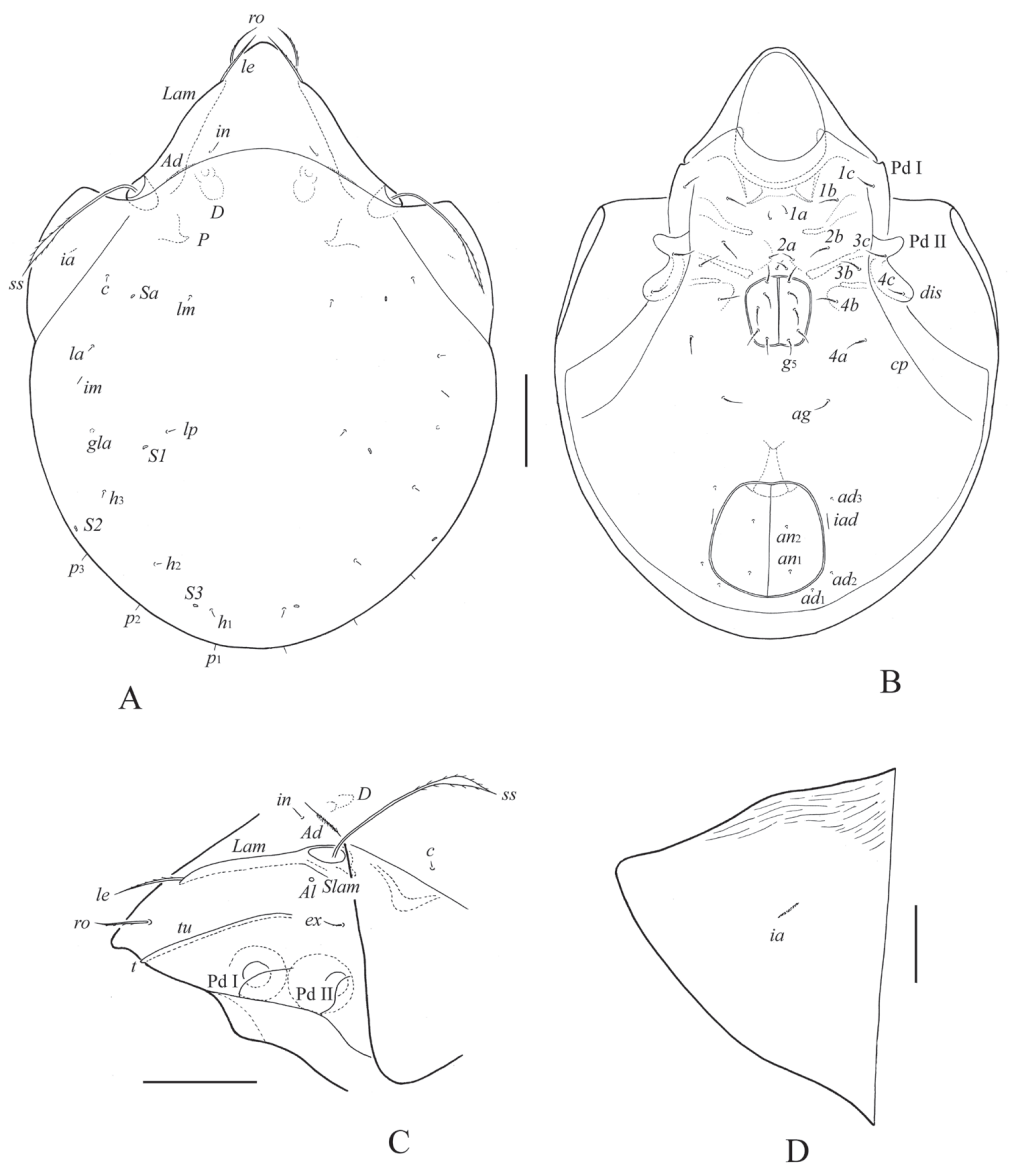
*Haplozetes paraminimicoma* sp. n., adult: **A** body dorsally **B** body ventrally (gnathosoma and legs not illustrated) **C** prodorsum and anterior part of notogaster laterally **D** left pteromorph. Scale bar (**A–C**) 50 μm, scale bar (**D**) 20 μm.

**Figure 2.**
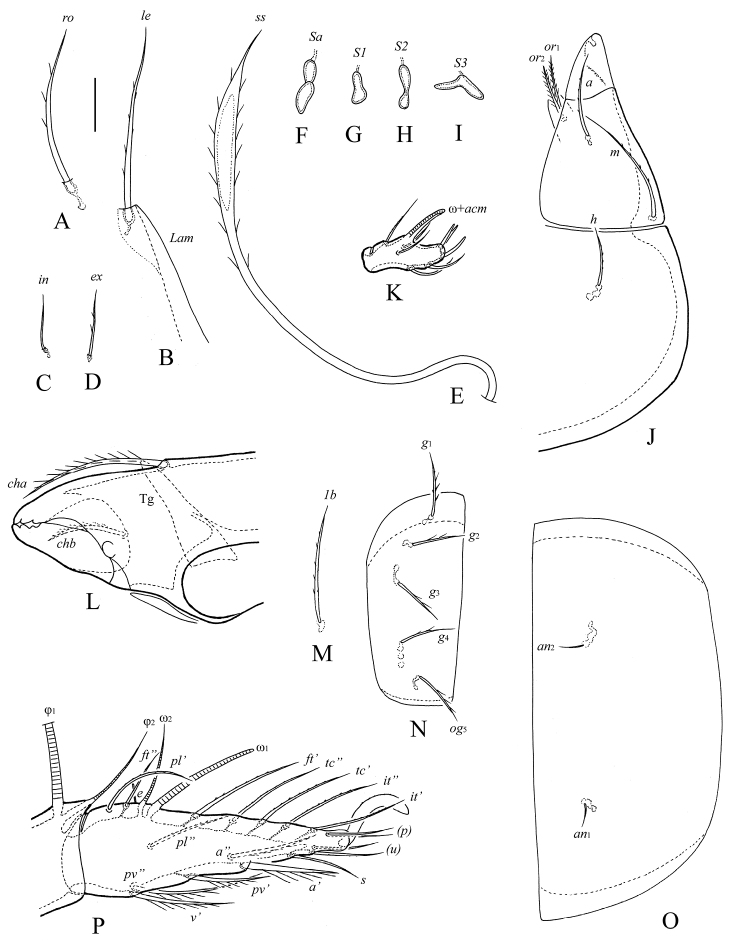
*Haplozetes paraminimicoma* sp. n., adult: **A** rostral seta **B** lamellar seta and anterior part of lamella **C** interlamellar seta **D** exobothridial seta **E** sensillus **F** sacullus *Sa* **G** sacullus *S1* **H** sacullus *S2* **I** sacullus *S3* **J** left half of subcapitulum **K** palptarsus **L** anterior part of chelicera **M** epimeral seta *1b* **N** right genital plate **O** left anal plate **P** tarsus and anterior part of tibia of leg I, left, paraxial view. Scale bar 10 μm.

#### Material examined.

Holotype (female) and four paratypes (two females and two males): Ec-1.

#### Type deposition.

The holotype (in ethanol) is deposited in the collection of the Zoological Institute of the Russian Academy of Sciences, St. Petersburg, Russia; two paratypes (in ethanol) are deposited in the collection of the Siberian Zoological Museum, Novosibirsk, Russia; two paratypes (in ethanol) are in the personal collection of the first author.

#### Etymology.

The prefix *para* is Latin meaning “near” and refers to the similarity between the new species and the species, *Haplozetes minimicoma* Beck, 1964.

#### Remarks.

In having the combination of main morphological characters (sensilli spindle-form, ciliate; interlamellar, notogastral and ano-adanal setae short; leg tarsi with one claw), *Haplozetes paraminimicoma* sp. n. is most similar to *Haplozetes minimicoma* Beck, 1964 from the Neotropical region and India (see Beck 1964), however the new species clearly differs from the latter by the presence of five pairs genital setae (versus four), smooth body surface (versus microfoveolate), long tutoria, extending insertions of rostral setae (versus not reaching), longer, ciliate genital and epimeral setae (versus short, smooth), and the absence of setae *l*’’ on leg tarsi I (versus present).

### Protoribates ecuadoriensis sp. n.

http://zoobank.org/B13B53C5-6850-4437-A493-5E7885A44D58

http://species-id.net/wiki/Protoribates_ecuadoriensis

Figs 3 , 4

#### Diagnosis.

Body size 547–647 × 332–431. Prodorsal setae long, setiform, barbed. Exobothridial setae minute. Sensilli with long stalk, lanceolate head and thin, point tip; distal part of stalk and sensillar head ciliate. Sublamellar porose areas large, oval. Notogastral porose areas of medium size, oval. Notogastral setae short. Adanal setae *ad*_1_ longer than *ad*_2_, *ad*_2_ longer than *ad*_3_.Legs monodactylous. Leg tarsi I, II with large dorsal tubercles. Tarsi I with 20 setae.

#### Description.

*Measurements*. Body length: 630 (holotype), 547–647 (five paratypes); notogaster width: 415 (holotype), 332–431 (five paratypes).

*Integument*. Body color light brownish to brown. Body surface microgranulate (visible only under high magnification).

*Prodorsum*. Rostrum rounded. Lamellae located dorso-laterally, not longer than half of prodorsum, hardly reaching insertions of lamellar setae. Rostral (49–57), lamellar (86–94) and interlamellar setae (123–135) setiform, barbed. A pair of elongate, narrow porose areas *Ad* present latero-posterior to interlamellar setae (visible under high magnification in dissected specimen). Exobothridial setae minute (4), thin, smooth. Sensilli (102–108) with long stalk, lanceolate head and thin, pointed tip; distal part of stalk and sensillar head ciliate. Tutoria short, narrow, slightly arched distally. Sublamellar lines short, very thin, straight, poorly visible. Sublamellar porose areas large, oval (20 × 16). Porose areas *Am* and *Ah* not observed.

*Notogaster*. Anterior notogastral margin convex medially. Dorsophragmata and pleurophragmata distinct. Pteromorphs sub-triangular. Ten pairs of notogastral setae short (4–6), thin, smooth. Four pairs of porose areas of medium size, oval: *Aa* (16–20 × 12–16) slightly larger than *A1*, *A2* and *A3* (10–14 × 8–12). Setae *lp* inserted posteriorly to *A1*. Lyrifissures (*ia*, *im*, *ip*, *ih*, *ips*) and opisthonotal gland openings located typically for the genus (see Weigmann et al. 1993; Miko et al. 1994). Postanal porose area absent.

*Gnathosoma*. Subcapitulum longer than wide (147–164 × 102–110). Subcapitular setae setiform, barbed; *h* and *a* (both 24–28) longer than *m* (10–14). Two pairs of adoral setae (16–20) setiform, barbed. Palps (90) with setation 0–2–1–3–9(+ω). Solenidion thickened, coupled with eupathidium. Chelicerae (147–164) with two setiform, barbed setae; *cha* (41–45) longer and thicker than *chb* (24–28). Trägårdh’s organ conical.

*Epimeral and lateral podosomal regions*. Apodemes 1, 2, 3 and sejugals well developed. Epimeral setal formula 3–1–3–3; setae setiform, slightly barbed. Medial setae *1a*, *2a*, *3a* (12–14) shorter than others (20–24). Pedotecta I, II, discidia and circumpedal carinae developed typically for the genus (see Weigmann et al. 1993; Miko et al. 1994). Custodia indistinct, widely blunt.

*Anogenital region*. Five pairs of genital (*g*_1_, 18–24, *g*_2_–*g*_5_, 12–16), one pair of aggenital (12), two pairs of anal (12) and three pairs of adanal (*ad*_1_, 22–24, *ad*_2_, 14–16, *ad*_3_, 12) setae setiform, slightly barbed. Lyrifissures *iad* in paraanal position.

*Legs*. All tarsi monodactylous. Morphology of leg segments, setae and solenidia typical for genus (see Weigmann et al. 1993; Miko et al. 1994), but tarsi I, II with large dorsal tubercles (*t*). Formulae of leg setation and solenidia: I (1–5–3–4–20) [1–2–2], II (1–5–3–4–15) [1–1–2], III (2–3–1–3–15) [1–1–0], IV (1–2–2–3–12) [0–1–0]; homology of setae and solenidia indicated in Table 2.

**Table 2.** Leg setation and solenidia of *Protoribates ecuadoriensis*, sp. n. (same data for *Trachyoribates (Rostrozetes) glaber*).

Leg	Trochanter	Femur	Genu	Tibia	Tarsus
I	*v*’	*d*, *(l)*, *bv*’’, *v*’’	*(l)*, *v*’, σ	*(l)*, *(v)*, φ_1_, φ_2_	*(ft)*, *(tc)*, *(it)*, *(p)*, *(u)*, *(a)*, *s*, *(pv)*, *v*’, *(pl)*, *l*’’, *e*, ω_1_, ω_2_
II	*v*’	*d*, *l*_1_’, *l*_2_’, *bv*’’, *v*’’	*(l)*, v’*, σ	*(l)*, *(v)*, φ	*(ft)*, *(tc)*, *(it)*, *(p)*, *(u)*, *(a)*, *s*, *(pv)*, ω_1_, ω_2_
III	*l*’, *v*’	*d*, *l*’, *ev*’	*l*’, σ	*l*’, *(v)*, φ	*(ft)*, *(tc)*, *(it)*, *(p)*, *(u)*, *(a)*, *s*, *(pv)*
IV	*v*’	*d*, *ev*’	*d*, *l*’	*l*’, *(v)*, φ	*ft*’’, *(tc)*, *(p)*, *(u)*, *(a)*, *s*, *(pv)*

See Table 1 for explanations. * – seta *v*’ absent in *Trachyoribates (Rostrozetes) glaber*.

**Figure 3.**
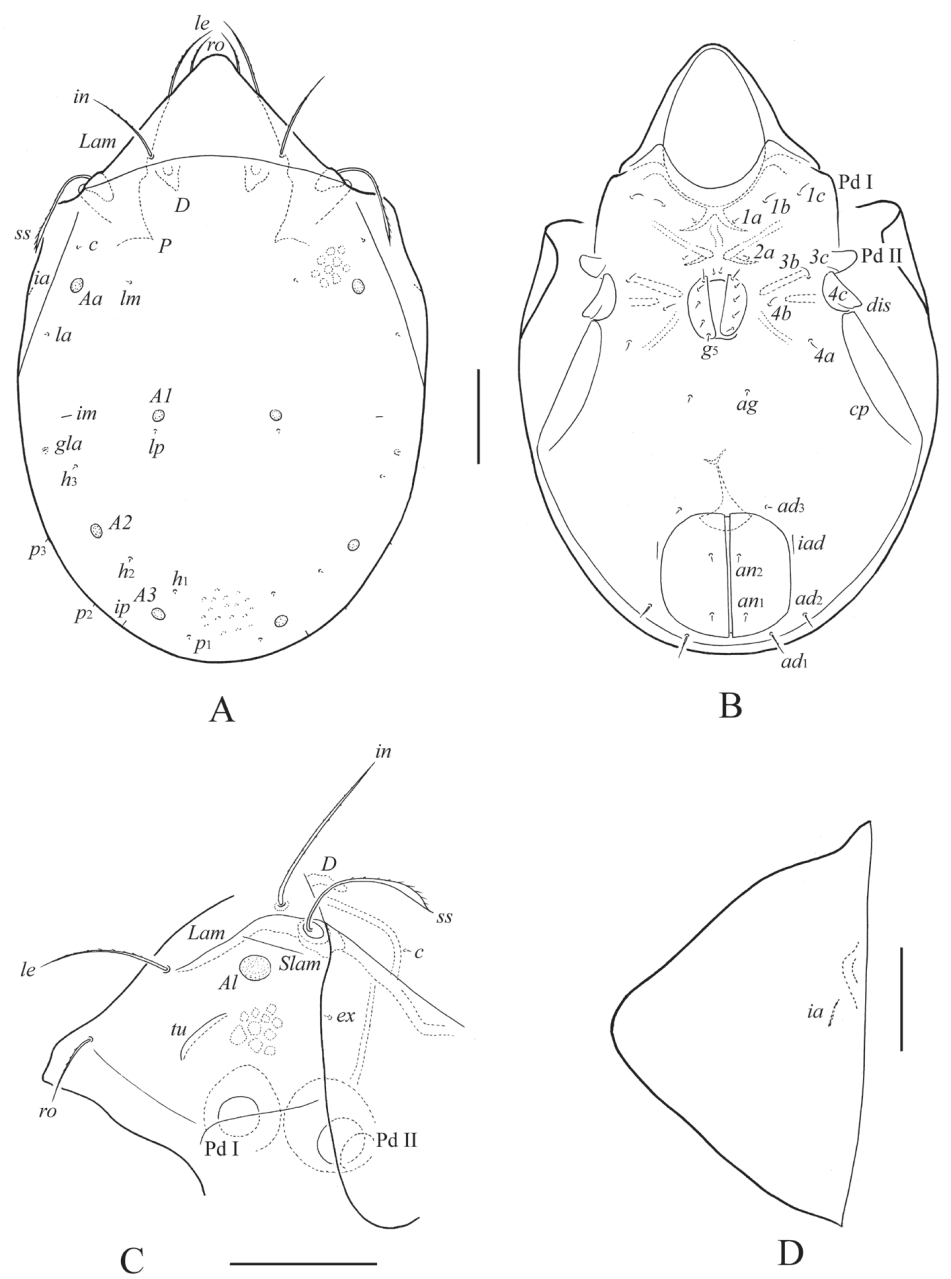
*Protoribates ecuadoriensis* sp. n., adult: **A** body dorsally **B** body ventrally (gnathosoma and legs not illustrated) **C** prodorsum and anterior part of notogaster laterally **D** left pteromorph. Scale bar (**A–C**) 100 μm, scale bar (**D**) 50 μm.

**Figure 4.**
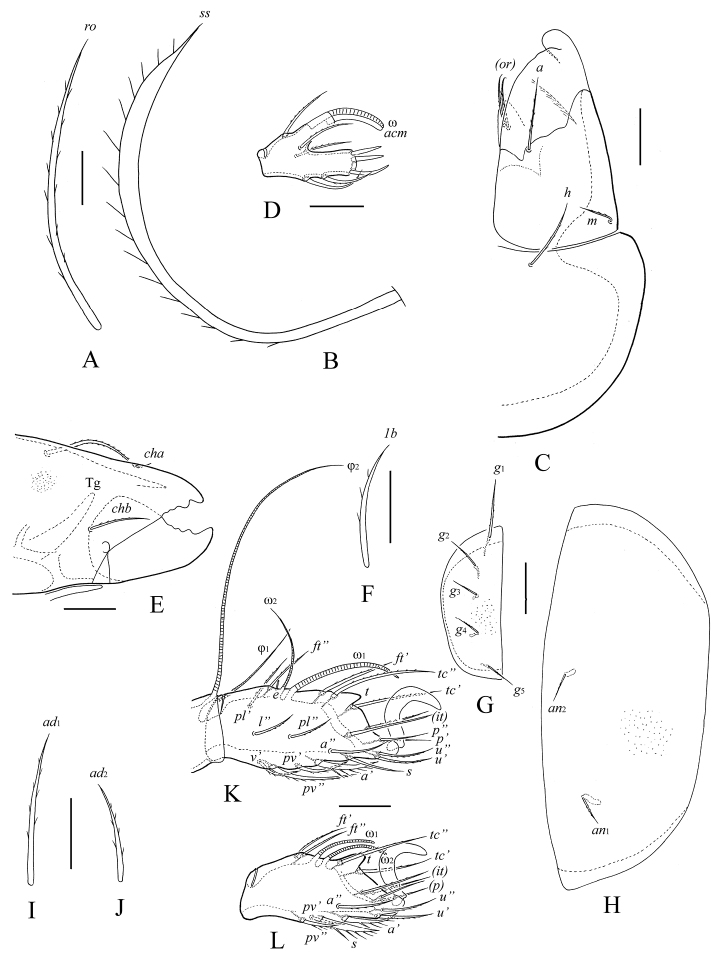
*Protoribates ecuadoriensis* sp. n., adult: **A** rostral seta **B** sensillus **C** left half of subcapitulum **D** palptarsus **E** anterior part of chelicera **F** epimeral seta *1b* **G** right genital plate **H** left anal plate **I** adanal seta *ad*_1_ **J** adanal seta *ad*_2_ **K** tarsus and anterior part of tibia of leg I, right, antiaxial view **L** tarsus of leg II, right, antiaxial view. Scale bar (**A, B, D, F, I, J**) 10 μm, scale bar (**C, E, G, H, K, L**) 20 μm.

#### Material examined.

Holotype (female) and five paratypes (two females and three males): Ec-1.

#### Type deposition.

The holotype (in ethanol) is deposited in the collection of the Zoological Institute of the Russian Academy of Sciences, St. Petersburg, Russia; three paratypes (in ethanol) are deposited in the collection of the Siberian Zoological Museum, Novosibirsk, Russia; two paratypes (in ethanol) are in the personal collection of the first author.

#### Etymology.

The specific name “*ecuadoriensis*” refers to the country of origin, Ecuador.

#### Remarks.

In having the combination of main morphological characters (monodactylous legs; body of medium size; prodorsal setae long, simple, barbed; sensilli long, with lanceolate, ciliate head; four pairs of porose areas oval; adanal setae *ad*_1_ longer than *ad*_2_, *ad*_2_ longer than *ad*_3_), *Protoribates ecuadoriensis* sp. n. is most similar to *Protoribates oblongus* (Ewing, 1909) from the Nearctic region (see Ewing 1909; Jacot 1937), however, the new species clearly differs from the latter by the presence of large tubercles on dorsal side of leg tarsi I and II.

### Supplementary description of a little-known species

#### Trachyoribates (Rostrozetes) glaber

(Beck, 1965)

http://species-id.net/wiki/Trachyoribates_glaber

Figs 5 , 6

##### Diagnosis.

Body size 307–365 × 199–232. Body surface foveolate. Rostral and lamellar setae of medium size, slightly barbed; interlamellar setae short, thin, smooth. Sensilli clavate; sensillar head with several barbs distally. Tutoria fused distally to prolamellar lines. Anterior notogastral margin regular convex. Notogastral setae of medium size, smooth. Postanal porose area present. Ventral setae short, smooth. Legs monodactylous.

##### Description.

*Measurements*. Body length: 307–365 (eight specimens); notogaster width: 199–232 (eight specimens).

*Integument*. Body color light brownish. Body surface foveolate (diameter of foveolae up to 4 on rostrum, up to 3 on notogaster and ventral side, up to 2 on medio-basal part of prodorsum). Foveolae located densely on prodorsum, but sparse on notogaster and ventral side. Also microgranules present on prodorsum.

*Prodorsum*. Rostrum rounded. Lamellae located dorso-laterally, longer than half of prodorsum, reaching insertion of lamellar setae. Prolamellar lines well developed. Rostral (32–41) and lamellar (49–57) setae setiform, slightly barbed. Interlamellar setae thin, smooth, shorter (28–32) and thinner than lamellar setae. A pair of elongate, narrow porose areas *Ad* present latero-posterior to interlamellar setae (visible only in dissected specimen). Exobothridial setae and their alveoli absent. Sensilli longest setae on prodorsum (61–73), with long stalk and clavate head; sensillar head with several barbs distally. Tutoria long, fused distally to prolamellar lines forming point tip (*t*), not reaching to insertions of rostral setae. Sublamellar lines short, thin, straight. Sublamellar porose areas small, rounded (4–8). Porose areas *Am* and *Ah* not observed.

*Notogaster*. Anterior notogastral margin regular convex. Dorsophragmata and pleurophragmata distinct. Pteromorphs sub-triangular. Notogastral setae represented by 10 pairs; they of medium size (*p*_1_–*p*_3_, 16–20; others, 24–32), thin, smooth. Four pairs of sacculi (*Sa*, *S1*, *S2*, *S3*) with small openings, but *S2* and *S3* visible only in dissected specimens. Lyrifissures and opisthonotal gland openings located typically for the genus (see Beck 1965). Postanal porose area oval (12 × 4).

*Gnathosoma*. Subcapitulum longer than wide (73–82 × 61–69). Subcapitular setae setiform, smooth; *h* (12) longer than *m* (6) and *a* (10). Two pairs of adoral setae (8) setiform, slightly barbed. Palps (41–45) with setation 0–2–1–3–9(+ω). Solenidion thickened, attached with eupathidium. Chelicerae (73–82) with two setiform, ciliate setae; *cha* (28–32) longer than *chb* (16–20). Trägårdh’s organ conical.

*Epimeral and lateral podosomal regions*. Apodemes 1, 2, 3 and sejugals well developed. Epimeral setal formula 3–1–3–3; setae short (4–6), setiform, smooth. Pedotecta I, II, discidia and circumpedal carinae developed typically for the genus (Beck 1965).

*Anogenital region*. Five pairs of genital (*g*_1_, 14, *g*_2_–*g*_5_, 6–8), one pair of aggenital (6–8), two pairs of anal (6–8) and three pairs of adanal (6–8) setiform, thin, smooth. Lyrifissures *iad* in paraanal position.

*Legs*. All tarsi with one strong, smooth claw. Formulae of leg setation and solenidia: I (1–5–3–4–20) [1–2–2], II (1–5–2–4–15) [1–1–2], III (2–3–1–3–15) [1–1–0], IV (1–2–2–3–12) [0–1–0]; homology of setae and solenidia indicated in Table 2.

**Figure 5.**
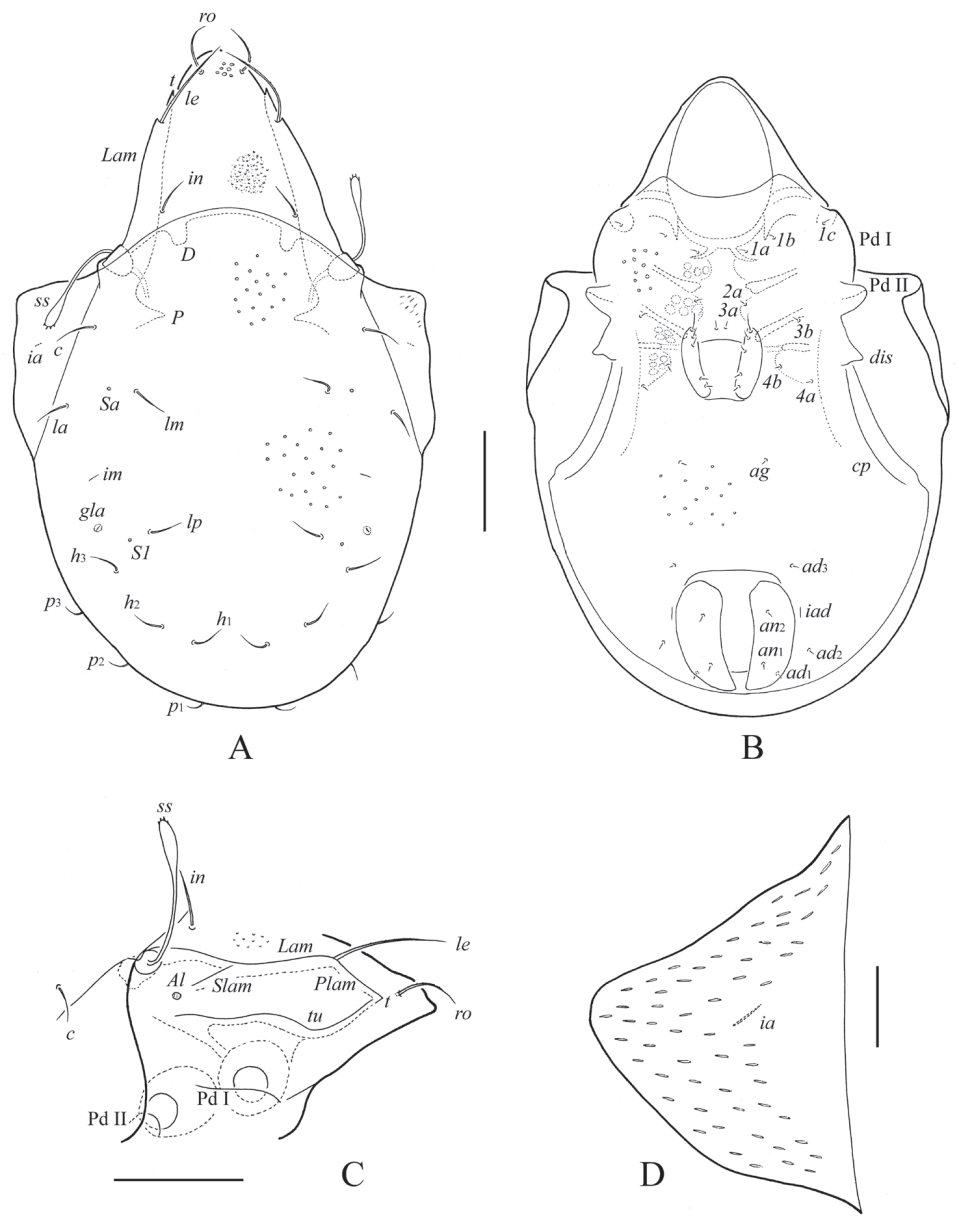
*Trachyoribates (Rostrozetes) glaber* (Beck, 1965), adult: **A** body dorsally **B** body ventrally (gnathosoma and legs not illustrated) **C** prodorsum and anterior part of notogaster laterally **D** left pteromorph. Scale bar (**A–C**) 50 μm, scale bar (**D**) 20 μm.

**Figure 6.**
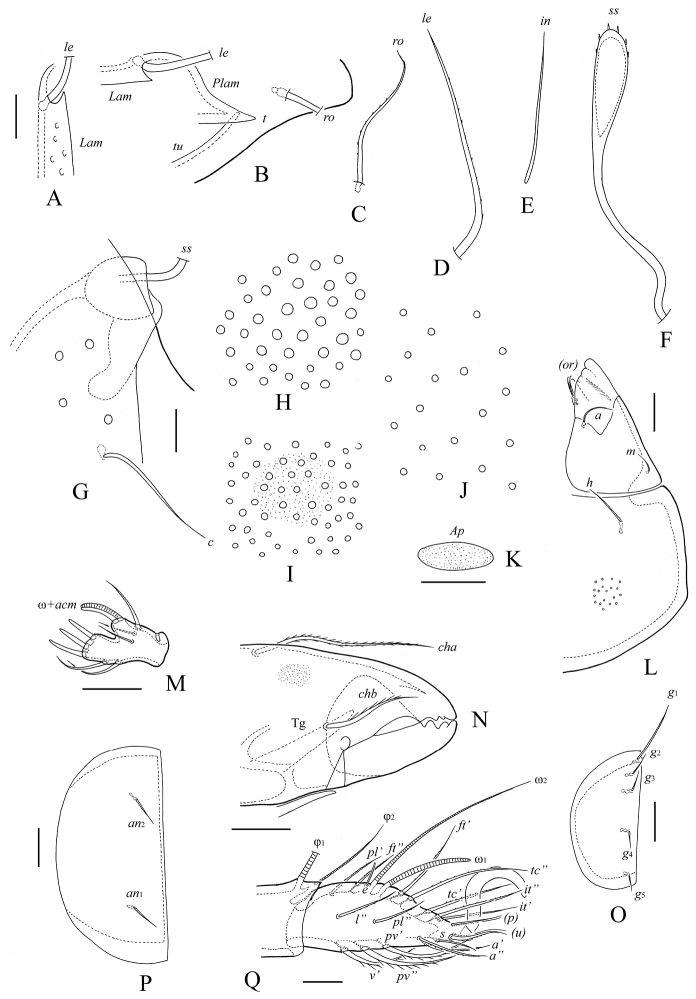
*Trachyoribates (Rostrozetes) glaber* (Beck, 1965), adult: **A** anterior part of lamella (medio-distal part of lamellar seta not illustrated) **B** anterior part of lamella and tutoria, and prolamellar line dorso-laterally (medio-distal part of rostral and lamellar seta not illustrated) **C** rostral seta **D** lamellar seta **E** interlamellar seta **F** sensillus **G** bothridium and notogastral seta *c* **H** foveolae on rostrum **I** foveolae in central part of prodorsum **J** foveolae on notogaster **K** postanal porose area **L** left half of subcapitulum **M** palptarsus **N** anterior part of chelicera **O** right genital plate **P** right anal plate **Q** tarsus and anterior part of tibia of leg I, right, antiaxial view. Scale bar 10 μm.

##### Material examined.

Eight specimens (five females and three males): Ec-1.

##### Remarks.

Actually the name of this species was first made available by Beck (1963) as *Rostrozetes glaber*, but its description was published later (Beck 1965). Judging on his brief description and illustrations, we identified our species as identical with *Trachyoribates (Rostrozetes) glaber*, known from Ecuador and Peru (see Beck 1965).

## Supplementary Material

XML Treatment for Haplozetes paraminimicoma

XML Treatment for Protoribates ecuadoriensis

XML Treatment for Trachyoribates (Rostrozetes) glaber
